# Recurrent EGFR alterations in NTRK3 fusion negative congenital mesoblastic nephroma

**DOI:** 10.1016/j.plabm.2020.e00164

**Published:** 2020-05-16

**Authors:** Li Lei, Bradley A. Stohr, Stacey Berry, Christina M. Lockwood, Jessica L. Davis, Erin R. Rudzinski, Christian A. Kunder

**Affiliations:** a Department of Pathology, Stanford University School of Medicine, Stanford, CA, USA; b Department of Pathology, University of California, San Francisco, San Francisco, CA, USA; c Department of Pathology, Cook Children’s Medical Center, Fort Worth, TX, USA; d Department of Laboratory Medicine, University of Washington, Seattle, WA, USA; e Department of Pathology, Oregon Health & Science University, Portland, OR, USA; f Department of Laboratories, Seattle Children’s Hospital, Seattle, WA, USA

**Keywords:** Congenital mesoblastic nephroma, *EGFR,* kinase domain duplication, *ETV6-NTRK3*, CCSK, clear cell sarcoma of the kidney, CMN, congenital mesoblastic nephroma, FISH, fluorescence in situ hybridization, IFS, infantile fibrosarcoma, ITD, internal tandem duplication, KDD, kinase domain duplication, NGS, next-generation sequencing

## Abstract

**Objectives:**

To identify oncogenic driver mutations in congenital mesoblastic nephroma (CMN) cases lacking *ETV6*-*NTRK3* fusion and discuss their diagnostic value.

**Design:**

The institutional pathology database was queried for cases with a morphologic diagnosis of CMN. Cases positive for *ETV6* rearrangement or with unavailable blocks were excluded. Four cases met the inclusion criteria and were sequenced by next-generation sequencing. Three additional cases were contributed by our collaborators.

**Results:**

Three of four internal cases harbor an *EGFR* kinase domain duplication (KDD), which is known to be oncogenic yet exceedingly rare in other histologies. All three outside cases are positive for *EGFR* alterations, including KDD in two and a splicing site mutation in one. The splicing site mutation is predicted to be EGFR activating. One of the outside cases was a retroperitoneal mass without a clear site of origin. A diagnosis of CMN is suggested based on exclusion of differential diagnoses by expert consultation and detection of *EGFR* KDD.

**Conclusions:**

EGFR activation, predominantly via *EGFR* KDD, is a common recurrent genetic alteration in CMN lacking *NTRK3* fusions. CMN can be molecularly classified into *NTRK3* fusion type, EGFR activation type and others.

## 1 Introduction

Congenital mesoblastic nephroma (CMN), the most common renal tumor of infancy, is a mesenchymal neoplasm histologically classified into classic, cellular, or mixed types. Most cellular CMNs harbor a characteristic *ETV6*-*NTRK3* fusion [[1], [2], [3], [4]]. A variant *EML4-NTRK3* fusion has also been described in rare cases [5]. The frequency of *NTRK3* fusions in the mixed type of CMN varies greatly by study [[2], [3], [4]]. For the classic subtype and the subset of cellular/mixed CMNs lacking *NTRK3* fusions, no recurrent genetic aberration had been identified, until a recently published series found *EGFR* kinase domain duplications (KDD), rare *NTRK1* fusions, and *BRAF* fusions and intragenic rearrangements [6].

The *EGFR* KDD has been described previously in rare cases of glioblastoma and lung adenocarcinoma [[7], [8], [9]]. It is an in-frame tandem duplication of exons 18–25, which encode the entire EGFR tyrosine kinase domain [8,9]. Intragenic tandem duplication is a well-known mechanism to activate oncogenes, for example, *FLT3* internal tandem duplication (ITD) in acute myeloid leukemia and *BCOR* ITD in clear cell sarcoma of kidney (CCSK). The oncogenic potential of the *EGFR* KDD has been observed in both cultured cells and patients [9]. We herein analyze a separate cohort and confirm that *EGFR* mutations, and in particular KDD, are important recurrent genetic alterations in many of these *NTRK3* fusion negative CMNs, and we discuss the clinicopathologic features of such cases in detail.

## 2 Materials and methods

### 2.1 Case selection

This study was triggered by the finding of an *EGFR* KDD in the index patient (case 1) during routine clinical testing. After obtaining IRB approval, the Stanford institutional pathology database was queried for cases with a morphologic diagnosis of CMN. Pathology reports and medical records were reviewed to document demographic data, histologic findings, treatment and outcome on the most recent follow up. Cases positive for *ETV6* rearrangement by fluorescence in situ hybridization (FISH) or t(12;15)(p13;q25) by conventional karyotype, or cases with unavailable blocks were excluded. Four cases (case 1 through 4) met the inclusion criteria, with the oldest being from 1996.

### 2.2 Next-generation sequencing and analysis

Formalin-fixed paraffin-embedded tissue was submitted for sequencing. Mutational profiling was performed using an institutionally-developed, hybrid capture-based next-generation sequencing (NGS) assay targeting 130 genes entirely or in part, which detects single nucleotide variants, short insertions and deletions, selected fusions, and selected amplifications in solid tumors, with tumor-only sequencing [10].

In addition, genome-wide copy number alterations were assessed in the four internal cases by generating off-target low-depth whole genome read plots, which are produced by counting the off target reads in each 1 megabase interval across the genome. These counts are then normalized and compared to a pool of normal diploid samples, with good concordance with conventional karyotyping in cases with high tumor content.

Three additional cases (cases 5, 6 and 7) contributed by our collaborators were analyzed by UCSF500 Cancer Gene Panel, Foundation One and UW Oncoplex, respectively.

### 2.3 Immunohistochemistry

Immunohistochemistry was performed following standard autostaining protocols. In brief, 4 ​μm sections prepared from the paraffin blocks were deparaffinized, rehydrated, and treated with 3% hydrogen peroxide for 15 ​min to quench endogenous peroxidase. After antigen retrieval, the slides were incubated with different primary antibodies, followed by incubation with a corresponding secondary antibody conjugated to horseradish peroxidase. Primary antibodies used are EGFR (5B7, prediluted, Ventana), WT1 (6F-H2, prediluted, Ventana/Cell Marque), h-caldesmon (h-CD, 1:25 dilution; Dako), smooth muscle actin (1A4, 1:200 dilution; Cell Marque), S100 (polyclonal, 1:1000 dilution; Dako), and CD99 (O13, prediluted, Ventana). Development was performed using a Bond Polymer Refine Detection system (Leica) and the 3,3′-diaminobenzidine chromogen. Appropriate positive and negative controls were included and evaluated with the specimens tested. EGFR staining was evaluated as to the subcellular pattern of staining, its intensity, and the percentage of cells staining. The other antibodies were evaluated as in routine clinical practice, with notes regarding the pattern and intensity of staining as appropriate.

## 3 Results

**Fig. 1:**
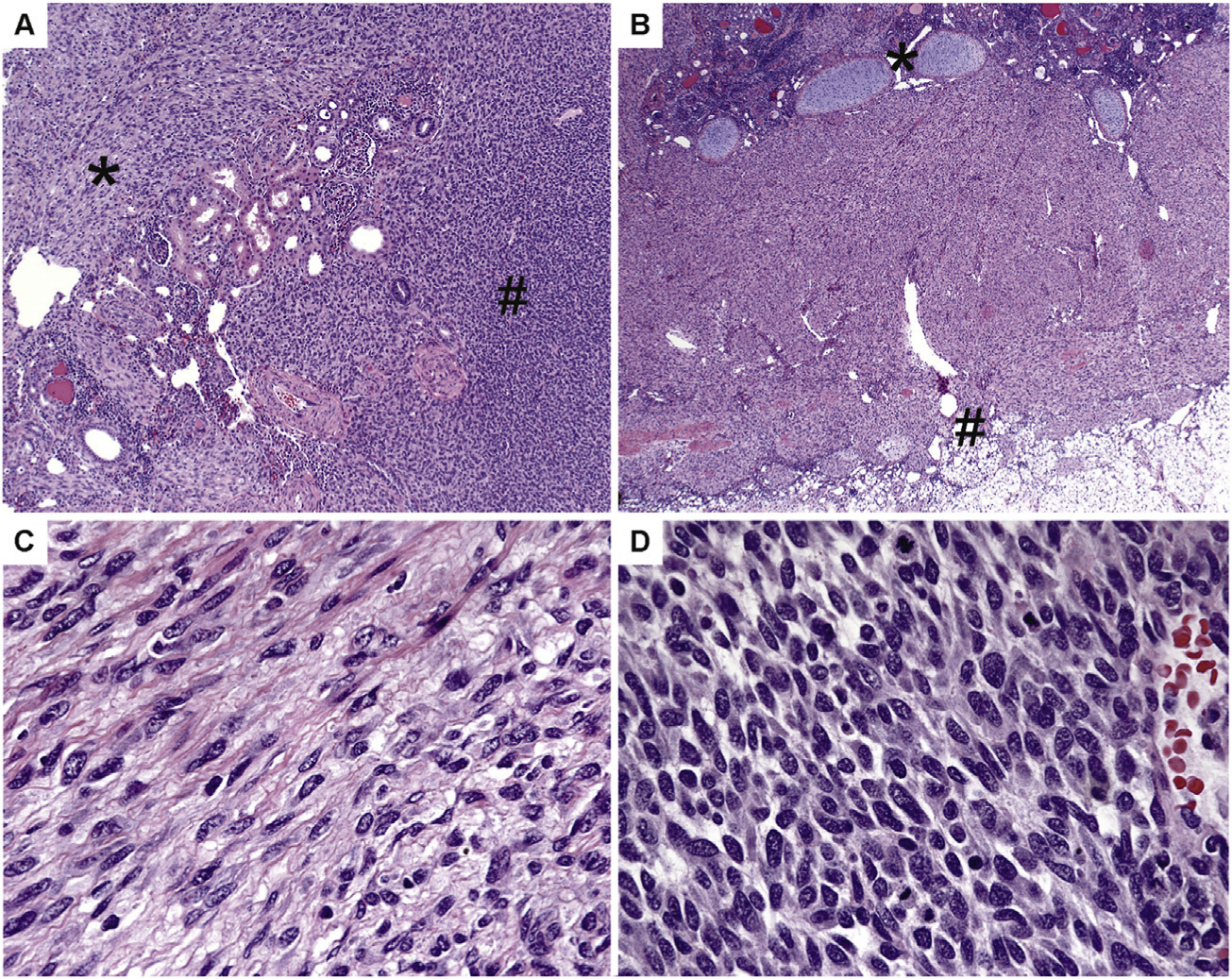
Histology of the index case. A, The tumor was comprised of two distinct components, hypopcellular (denoted by ∗) and hypercellular areas (denoted by #). Entrapped tubules were frequently encountered (center). B, At the edges of the tumor, the spindle cell component extended into the surrounding parenchyma or perinephric fat (denoted by #). Small nodules of mature cartilage (denoted by ∗) were present. C, The hypocellular areas showed fascicles of spindle cells with only very mild atypia in the form of slight nuclear membrane irregularities. The nuclei had speckled to vesicular chromatin. The cells had indistinct, tapering, eosinophilic cytoplasm. Occasional mitotic figures were identified (less than one per ten 0.1 ​mm^2^ high power fields). D, The hypercellular areas were composed of almost round cells, with considerably less spindling, and a markedly higher mitotic rate (up to twelve per ten 0.1 ​mm^2^ high power fields) in excess of the greater cellularity. The cytomorphology was similar otherwise.

The index patient was a 6-week-old boy with a 4.1 ​cm, poorly-circumscribed renal tumor. Microscopically, the tumor was comprised of two distinct components (Fig. 1A). The largest component showed bland spindle cells in broad, intersecting fascicles. At the edges of the tumor, this component extended irregularly and extensively into the surrounding parenchyma in a plexiform manner, and entrapped tubules were frequently encountered. The tumor seemed to have an affinity for the renal capsule, extending along it in places, and infiltrating through it into the surrounding perinephric fat. Small nodules of mature cartilage were present in some areas at the interface between this capsular component and the more central renal parenchyma (Fig. 1B). The cells showed only very mild atypia in the form of slight nuclear membrane irregularities. The nuclei had speckled to vesicular chromatin. The cells had indistinct, tapering, eosinophilic cytoplasm. Numerous mast cells were scattered throughout. Occasional mitotic figures were identified (less than one per ten 0.1 ​mm^2^ high power fields) (Fig. 1C). A sharply demarcated second component was also present. These other areas were composed of highly cellular, almost round-cell areas, with considerably less spindling. The cytomorphology was similar otherwise, and these areas were notable for a markedly higher mitotic rate (up to twelve per ten 0.1 ​mm^2^ high power fields) in excess of the greater cellularity (Fig. 1D). Both components showed a vasculature consisting of wide, thin-walled, gaping, branching vessels resembling the vasculature of solitary fibrous tumor. These vessels were much more prominent in the cellular component. Immunohistochemistry showed that the tumor cells were positive for WT1 (strong and diffuse nuclear and cytoplasmic), caldesmon, CD99 (cytoplasmic, not membranous), S100 (subset), and SMA (subset). Overall, the findings were most consistent with CMN, mixed type.

**Fig. 2:**
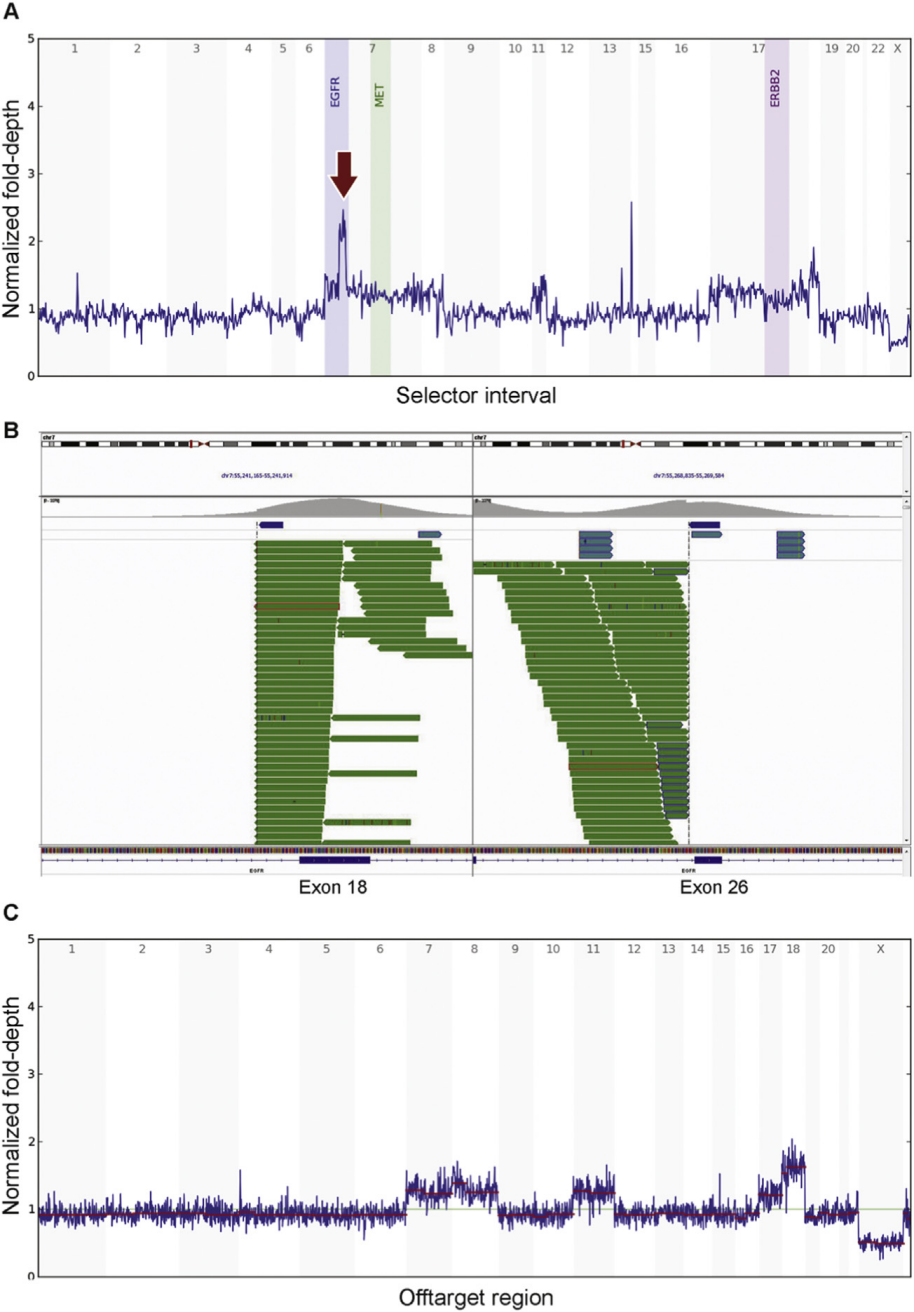
Molecular findings of the index case. A, Depth plot showing *EGFR* amplification as indicated by the arrow; B, IGV view showing *EGFR* KDD, with the upstream breakpoint located within intron 17 and the downstream breakpoint within intron 25; C, Off-target depth plot showing gain of chromosomes 7, 8, 11, 17 and 18, concordant with the conventional karyotyping result of 52,XY,+7,+8,+11,+17,+18,+18 [11]/46,XY [9] in this case.

FISH for *ETV6* rearrangement was negative in this case. Karyotyping showed trisomy 7, 8, 11, 17 and tetrasomy 18. Broad mutational profiling by targeted NGS was performed. By the standard clinical pipeline, no tumor-specific genetic alterations were identified except equivocal amplification of *EGFR* (Fig. 2A). The apparent increased depth for exons 18 to 25 above the rest of the gene prompted a manual review of the sequencing data, which revealed *EGFR* KDD (Fig. 2B), that is, a tandem duplication of exons 18 to 25. The abnormal genome-wide copy number assessment was concordant with the conventional karyotyping result (Fig. 2C).

**Fig. 3:**
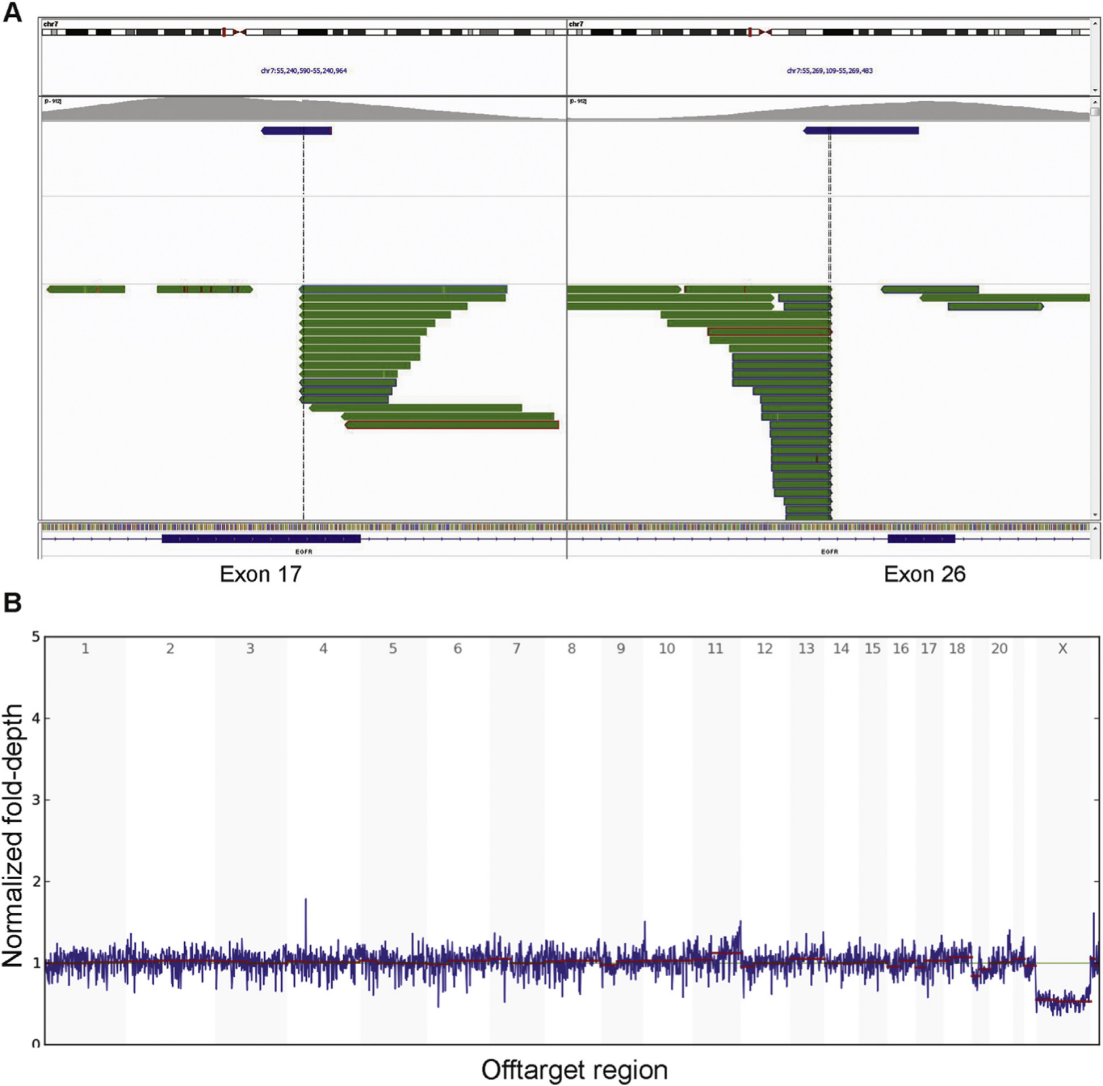
Molecular findings of case 2. A, IGV view showing *EGFR* KDD. The upstream breakpoint sits on exon 17 without disrupting the reading frame. The downstream breakpoint is located within intron 25. B, Off-target depth plot showing normal pattern, concordant with normal karyotype in this case.

An additional three cases from our institution without demonstrated *ETV6-NTRK3* fusions were then subjected to mutational profiling using the method described above. *EGFR* KDD was identified in two of these three cases (variant details in Supplemental Table 1). Breakpoints in these cases were located within introns 17 and 25 of *EGFR* gene, with one exception in case 2. The upstream breakpoint in case 2 was located within exon 17 (Fig. 3A). It is likely that this would result in skipping of the fragment of exon 17, and the result would again be a duplication of exons 18–25. A normal genome-wide copy number assessment of case 2 was also shown for comparison (Fig. 3B). The absence of *NTRK* fusions in the internal cases was further confirmed by manual review of the pileups, although the assay does not detect all *NTRK* fusions due to coverage limitations (all three genes are covered, but some introns potentially harboring genomic breakpoints are not completely covered). The only internal case with no oncogenic mutations identified, case 3, is distinct from others in that an expert consultation is needed to establish the diagnosis because of the unusual plump cell morphology with necrosis as well as older age at presentation (see Table 1 for details).

**Table 1:** Clinicopathologic and genetic findings of *NTRK3* fusion negative CMNs.

Case	Type	Age at diagnosis	Sex	Size (cm)	ETV6 FISH	EGFR	Treatment	Duration of follow-up (months)	Outcome
1	Mixed	6 weeks	M	4.1	–	KDD	Nephrectomy	48	NED
2	Mixed	Prenatal	M	7.6	–	KDD	Nephrectomy	90	NED
3	Cellular^a^	4 years	M	6.5	N/A	–	Nephrectomy and chemotherapy	128	NED
4	Classic	Prenatal	M	9	N/A	KDD	Nephrectomy	106	NED
5	Classic	7 days	M	5.5	N/A	KDD	Nephrectomy	24	NED
6	Mixed (Predominantly cellular)	5 months	M	8.5	N/A	Splicing site mutation	Nephrectomy	N/A	N/A
7	Retroperitoneal mass^b^	1 year	M	16.5	–	KDD	Debulking and chemotherapy	48	Residual disease

M indicates male; -, negative; N/A, not available; NED, no evidence of disease.

# See Supplemental Table for details of genetic findings.

a Due to the unusual plump cell morphology with necrosis and older age at presentation, the diagnosis was confirmed by expert consultation.

b The case was a retroperitoneal complex cystic mass that involved the left kidney. It was initially signed out as undifferentiated spindle cell sarcoma. Retrospectively, the morphologic features are compatible with CMN, mixed type.

Another two CMN cases with *EGFR* alterations were contributed by our collaborators. One of these (case 5) also showed the *EGFR* KDD. Interestingly, case 6 harbors a splice site mutation in *EGFR*. This splice site mutation involves a canonical donor splice site of intron 26 and is predicted to cause exon 26 skipping, and consequent EGFR activation based on the current knowledge on the function of the carboxyl-terminus of EGFR.

**Fig. 4:**
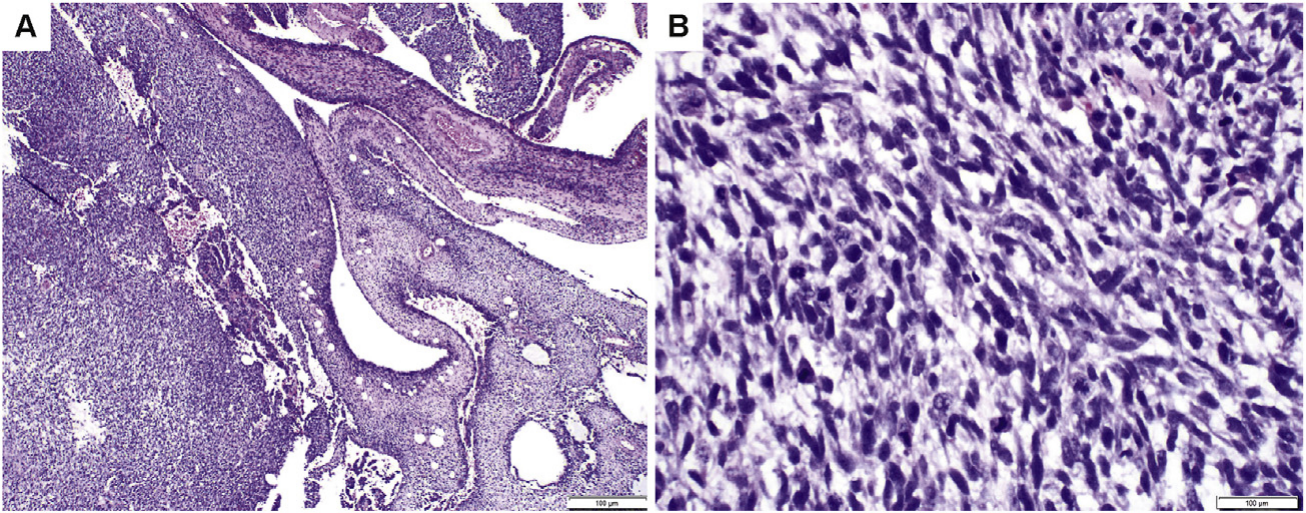
Histology of case 7. A. The tumor was a variably cellular spindle cell neoplasm forming cysts and septa, with a dense fascicular appearance in some areas. The cells appeared to condense around the cystic spaces. B. Tumor cells exhibited oval hyperchromatic nuclei with small nucleoli, and indistinct cytoplasm. Mitotic activity was enumerated at greater than forty per ten high power fields.

Case 7 is clinically and histologically unique in our series. The patient presented with a 16.5 ​cm, multiseptated, largely complex cystic mass involving the lower pole of the left kidney. The histologic sections demonstrate a variably cellular spindle cell neoplasm that forms cysts and septa (Fig. 4A). Tumor cells exhibit oval hyperchromatic nuclei with small nucleoli, indistinct cytoplasm and numerous mitotic figures (greater than 40 per 10 high-power fields) (Fig. 4B). In some areas the tumor is myxoid. Other areas have a dense fascicular appearance. The cells appear to condense around the cystic spaces (Fig. 4A). Hemorrhagic and necrotic tissue comprises approximately 30% of the tumor. The case was initially signed out as undifferentiated spindle cell sarcoma. Of note, the differential diagnosis included *BCOR*-associated sarcoma based partially on a positive BCOR immunostain, and *NTRK*-related sarcoma as there was weak, patchy cytoplasmic staining for panTrk as well as moderate to strong diffuse cytoplasmic staining for TrkA. However, FISH for *BCOR, NTRK3,* as well as *SYT* gene rearrangements was negative. NGS identified an *EGFR* KDD but no evidence of *BCOR* ITD or an *NTRK* gene rearrangement. In addition to the *EGFR* mutation, a subclonal activating mutation in *PIK3CA* and a loss-of-function mutation in *ARID1A* were also identified. Conventional karyotyping revealed +8,+11,+17,+19,der(20)t(1;20)(q12;q13.2). The patient underwent debulking surgery, and completed multiple cycles of chemotherapy followed by extensive surgery as well as intratumoral bed cisplatin administration. At 14-month follow-up, pathology specimens showed multifocal viable tumor, and as of 48 months, he is undergoing additional chemotherapy.

The clinicopathologic and genetic findings of all the cases are summarized in Table 1 (with additional details in Supplemental Table 1). All patients except case 7 had a favorable outcome with no evidence of recurrent or metastatic disease, although there was limited follow-up (24–128 months, median of 48 months). Except for the subclonal *PIK3CA* activating mutation and an *ARID1A* loss-of-function mutation found in case 7, all other genetic alterations identified are variants of unknown significance and likely represent rare germline variants.

**Fig. 5:**
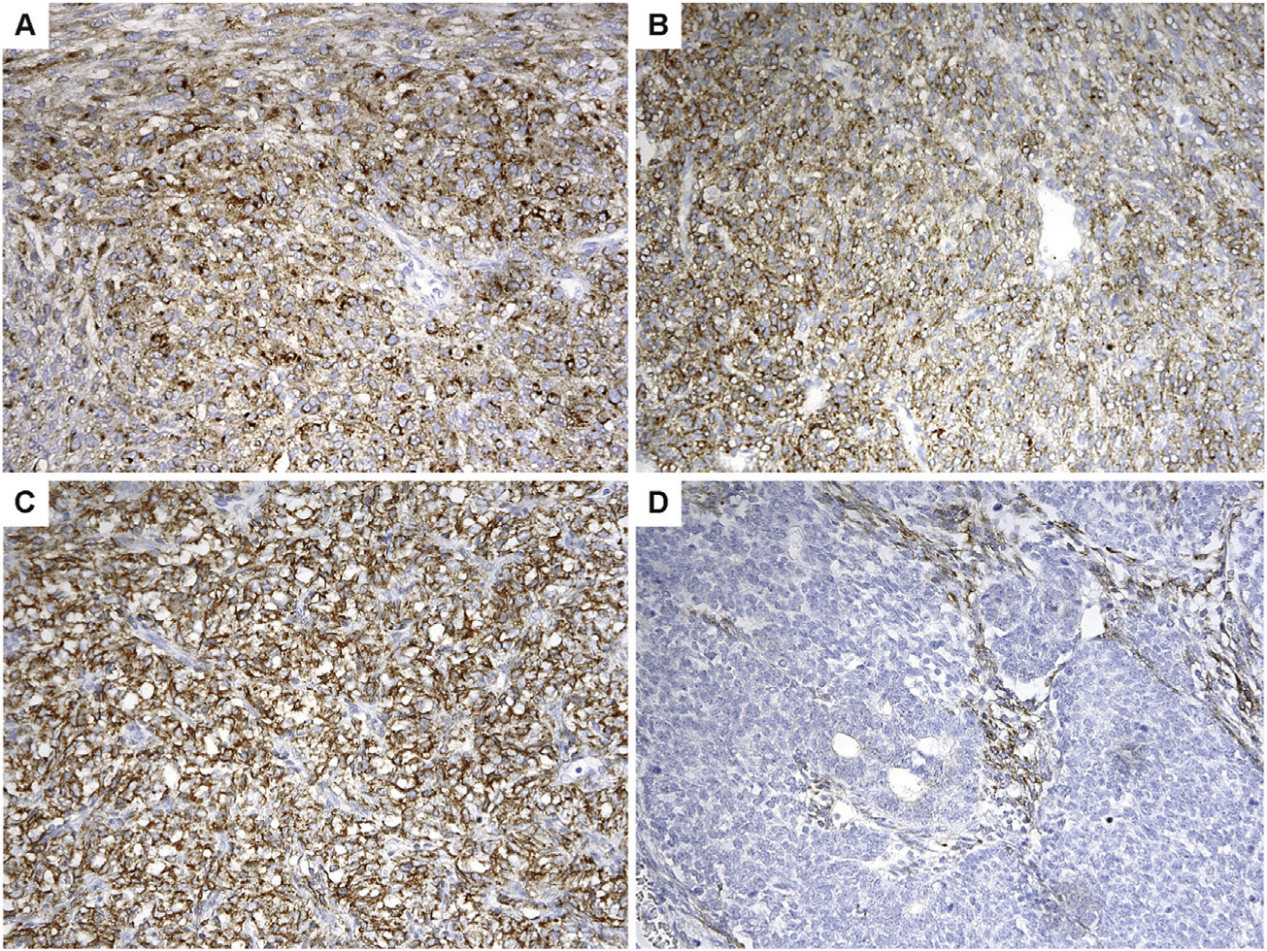
Different EGFR immunostaining patterns in CMN, CCSK and Wilms’ tumor. A, CMN with *EGFR* KDD demonstrated prominent dot-like Golgi reactivity (center) as well as very faint staining (top and lower left corner). B, CMN with *ETV6*-*NTRK3* showed immunoreactivity in a Golgi pattern, similar to that seen in CMN with *EGFR* KDD. C, CCSK showed diffuse, strong membranous reactivity. D, Wilms’ tumors blastemal elements were negative, but epithelial elements sometimes showed weak staining, and staining of unclear significance was also noted in stromal components surrounding tumor nests. (Immunohistochemistry, 200X).

To determine whether EGFR protein expression might be used as a surrogate marker to identify cases with EGFR *KDD*, we performed immunohistochemical staining on three CMNs harboring *EGFR* KDD, one CMN known to carry *ETV6-NTRK3* fusion, two cases of CCSK, and three cases of Wilms’ tumor. Staining was variable in the *EGFR* KDD cases, with some cases showing prominent dot-like Golgi reactivity while others showing only faint labeling (Fig. 5A). The one *ETV6-NTRK3* case stained also showed EGFR immunoreactivity in a Golgi pattern (Fig. 5B). The two CCSK cases each showed strong membranous reactivity (Fig. 5C). The Wilms’ tumor blastemal elements were negative, but epithelial elements sometimes showed weak staining, and staining of unclear significance was also noted in stromal components surrounding tumor nests (Fig. 5D).

## 4 Discussion

We report *EGFR* KDD as a recurrent oncogenic driver of CMN, alternative to *NTRK3* fusions. Our findings importantly corroborate those of a recent paper from Wegert et al., which found *EGFR* KDD in 30 of 45 classic CMNs, 11 of 15 mixed CMNs, and 2 of 20 cellular CMNs [6]. This alteration is referred to in that publication as *EGFR* ITD. Although this terminology is technically correct, we prefer the more widely-accepted term KDD for this very distinctive variant [[7], [8], [9]], as other unrelated ITDs with different activation mechanisms are known to occur in *EGFR* [11].

*EGFR* KDD, as the name implies, is a duplication of the kinase domain. However, variants of *EGFR* KDD can occur [7], as seen in case 2. Considering the in-frame nature and the duplication of the intact kinase domain, the reported variants are predicted to be functionally similar to prototypical *EGFR* KDD. Additional functional significance of the extra sequences, if any, remains uncertain.

Reliable detection of variants like *EGFR* KDD by NGS is a known challenge, as discussed in the literature [9]. In fact, the current clinical bioinformatics pipeline for the STAMP assay will not automatically detect this kind of variant, and the breakpoints in these cases were determined by manually reviewing the reads. The initial case was identified only because the depth (plotted by region for amplification calling) was noticeably higher in these exons of *EGFR* due to apparent concurrent amplification of the mutated allele.

Though the frequency of *EGFR* KDD may be underestimated due to the technical difficulty, it has only been reported in a limited number of entities since the initial description in 1998, with the reported highest frequency being 0.2% in lung adenocarcinoma [7,8]. Among the >38,000 cases sequenced at Foundation One, *EGFR* KDD was detected in 5 out of approximately 7200 lung cancers (0.07%), 3 gliomas, 1 soft tissue sarcoma, 1 peritoneal serous carcinoma and 1 Wilms’ tumor [9]. The surprisingly high frequency of *EGFR* KDD in CMN makes it a potentially highly specific molecular marker of the entity. In diagnostically difficult cases, detecting *EGFR* KDD may serve as an ancillary tool to help rule in or rule out the diagnosis.

Case 7 in our series represents such an example. The case was a large retroperitoneal tumor that involved the kidney of an infant. It was initially signed out descriptively as undifferentiated spindle cell sarcoma, with differential diagnosis including *BCOR*-associated sarcoma and *NTRK*-related sarcoma. Further workup showed the tumor to be negative for *ETV6* and *BCOR* gene rearrangement by FISH, and negative for *BCOR* ITD and *NTRK* gene rearrangement by NGS. Additionally, a conventional karyotyping showed +8,+11,+17,+19, der(20)t(1;20)(q12;q13.2). Polysomies, particularly trisomy 11 and less commonly polysomy 8, 17 and 20, are recurrent cytogenetic findings in CMN and infantile fibrosarcoma (IFS) [12,13]. The differential diagnosis was then narrowed down to CMN, IFS if not arising in the kidney, or a not otherwise specified spindle cell sarcoma. IFS is known to resemble cellular-type CMN histologically, as well as genetically, with both showing the *ETV6-NTRK3* fusion. Thus far, *EGFR* KDD has not been observed in IFS, including 27 negative cases in the Wegert series [6].

If it is demonstrated that a subset of IFS cases carries the *EGFR* KDD, this will only provide more support for the idea that biologically the two entities are the same thing arising in different locations. If *EGFR* mutations are only seen in CMN in this differential, then the presence of this alteration would be useful to separate the two tumors in cases like this where the site of origin is obscure.

It must be allowed, however, that some other findings in case 7 are atypical for CMN, including some morphologic features and the tumor’s clinical aggressiveness as shown by the progression following surgery and chemotherapy. Although generally considered indolent, rare cases of definite CMN are clinically aggressive and even have metastatic potential [[14], [15], [16]]. The genetic and morphologic commonalities between this tumor and CMNs, as well as the clinical context and possible origin in the kidney, all suggest that the spectrum of this disease may be wider than previously thought, and some poorly-differentiated abdominal neoplasms in infants may in fact be related etiologically to *EGFR*-mutated CMN, similarly to how *ETV6-NTRK3* positive CMN is proposed to be related to IFS. A relationship between the tumor in case 7 and CMN seems likely given the incredible rarity of these mutations in other settings, but additional work will be required to determine whether this is true.

In addition, as discussed above, a subclonal activating *PIK3CA* mutation and a pathogenic *ARID1A* mutation were also detected in case 7. In the largest comprehensive study to date, which involved whole genome sequencing of 10 *NTRK3* fusion-negative cases of CMN, no other pathogenic mutations were reported, including in *PIK3CA* and *ARID1A* [6]. No pathogenic *PIK3CA* mutations and three pathogenic *ARID1A* mutations (two of which were germline) were identified in a whole genome sequencing study of 117 cases of Wilms’ tumor [17]. To our knowledge, recurrent mutations in these genes have not been described in other pediatric soft tissue tumors, including CCSK and IFS, although in each case the number of cases examined comprehensively is currently very small. Thus, while these mutations may contribute to pathogenesis in this particular tumor, they do not appear to be highly recurrent events in CMN or related tumors.

Interestingly, *EGFR* KDD has recently been reported in a 5-month-old male with CCSK [18], another type of pediatric renal tumor usually driven by *BCOR* ITD, *BCOR*-*CCNB3* fusion or *YWHAE-NUTM2* fusion [19], but none of which was detected in this case. Classification of these primitive renal tumors remains difficult in cases without definitional molecular alterations. Given several features unusual for CCSK, we wonder if this case might actually be a CMN. Clinically, CCSK is extremely rare in infants younger than 6 months, whereas CMN is the most common renal tumor in infancy. In addition, CCSK is notorious for bone metastasis, which the patient did not have. Histologically, CCSK-like areas can be encountered in cases of CMN [20]. Immunohistochemically, nuclear expression of cyclin D1 was used to support the diagnosis of CCSK in this case. However, nuclear expression of cyclin D1 has been observed in CMN by multiple independent groups [[21], [22], [23]]. In one study, all cases of classic CMN demonstrated strong and diffuse nuclear expression of cyclin D1; Five out of six cases of mixed CMN were also positive [22]. Last but not least, cytogenetically, karyotyping showed gains of chromosomes 2, 5, 7, 8, 9, 11, 12, 17, 19, 20, and 21, typical of CMN but not CCSK [12,13].

The single case of *EGFR* KDD positive Wilms’ tumor in Gallant’s paper is also intriguing [9]. Though no details are available in this case, CMN may be mistaken as monophasic Wilms’ tumor, or as biphasic Wilms’ tumor when entrapped tubules mimic an epithelial element, a known diagnostic pitfall. In fact, no *EGFR* KDD was detected in 20 cases of CCSK and 208 cases of Wilms’ tumor in Wegert’s paper [6]. These cases stress the need for additional diagnostic tools to render correct diagnosis [9,18], as the clinical approach to these entities is markedly different. Patients with Wilms’ tumor may need chemotherapy and radiotherapy, depending on the stage of disease. In contrast, CMN can usually be cured with surgical resection. That said, inoperable or metastatic cases of CMN have been reported [[14], [15], [16],^24^]. These patients may benefit from genetic alteration-specific targeted therapies, such as larotrectinib or merestinib in cases with *NTRK* fusions [[24], [25], [26]], or potentially EGFR-directed therapies for cases carrying *EGFR* KDD [9,27]. In this study, immunohistochemistry for EGFR did not distinguish CMNs with *EGFR* KDD from those with *ETV6-NTRK3* fusion. Interestingly, CCSK also expressed EGFR; however, it demonstrated a divergent staining pattern which might be useful diagnostically, finding in accordance with previously reported result [28]. Wilms’ tumors seem to be mostly negative for EGFR expression. Confirming this will require additional study.

A novel finding of our study is that EGFR activation may occur via alternative mechanisms in CMN cases lacking *EGFR* KDD. The splicing site mutation identified in case 7 is predicted to cause *EGFR* exon 26 skipping, or a potentially larger deletion involving more downstream exons. The carboxyl-terminus of EGFR functions as an autoinhibitory domain through multiple mechanisms, such as binding to c-Cbl with consequent ubiquitination and lysosomal sorting for EGFR degradation, and palmitoylation to downregulate EGFR signaling [[29], [30], [31]]. Mutations involving palmitoylation sites within exon 26 promoted cell migration and transformation in an *in vitro* study [31]. A separate study showed exons 25 and 26-deleted *EGFR* mutant led to EGFR activation and cytokine independent proliferation [32]. Various carboxyl-terminal deletion mutants have been reported to be tumorigenic and, more importantly, sensitive to EGFR-directed therapies [33]. Therefore, the *EGFR* splicing site mutation found in case 7 is likely to cause EGFR activation via exon 26 skipping and may have potential therapeutic significance.

In the Wegert study, 10 of 10 classic CMNs studied by whole-genome sequencing contained an *EGFR* KDD, but only 20 out of 35 classic cases tested by RT-PCR were positive [6]. While Wegert et al. suggested some cases could be explained by less recurrent rearrangement variants of *BRAF*, *NTRK1* or *NTRK3* [6], our data show that the breakpoints of *EGFR* KDD are not invariant, and non-duplication activating mutations such as some splice site mutations can occur, both of which could also explain a false negative RT-PCR result. More cases are needed to clarify the frequency of *BRAF* or *NTRK1* alterations in non-*NTRK3*, non-*EGFR* CMNs.

The only case in our series with no pathogenic mutations identified, case 3, is distinct from others in terms of age at presentation and morphology. The diagnosis of this case was established by expert consultation and supported by the karyotyping results. As the only case of cellular CMN in our series, it was included because the possibility of an *NTRK3* fusion had not yet been tested by either FISH or conventional cytogenetics and therefore it did not meet the exclusion criteria. Nevertheless, no *NTRK3* fusion was identified by NGS, although the assay does not detect all *NTRK* fusions due to coverage limitations. Interestingly, *BRAF* fusions have recently been reported in a subset of *NTRK* fusion negative pediatric sarcomas showing morphologic overlap with IFS [34]. The clinical features of these *BRAF* fusion positive cases include older age at presentation and a predilection for axial location. However, manual review of the sequencing data failed to detect either *BRAF* or *NTRK* fusion in case 3.

It appears that immunohistochemistry for EGFR does not distinguish cases with *EGFR* KDD from *ETV6-NTRK3* CMNs. Although clear cell sarcoma of the kidney also appears to be EGFR positive, the staining pattern is different, which might be useful diagnostically, but the number of cases tested is very small, making definitive conclusions difficult. Wilms’ tumors seem to be mostly negative, in accordance with previously reported results [28].

Whether or not the *EGFR* KDD has any particular clinical associations, prognostic or otherwise, is not clearly known at this point. Poor outcomes are so rare in this disease (<10%) that many series of this rare tumor do not have any recurrences or metastases. However, aggressive behavior has been reported in every histologic subtype, primarily recurrence but including metastasis and death. The largest series of 111 cases with clinical data had a five-year recurrence free survival (RFS) of 93% [35]. As in other studies, recurrence risk was related to stage. In their population, the *ETV6-NTRK3* positive subset of cellular CMN had an excellent prognosis, with no recurrences in this group of 17 cases (versus an RFS of 73% for fusion-negative cellular CMNs). Histologic type was associated with recurrence risk also, with almost uniformly positive outcomes for cases with classic histology, but there were no significant differences in overall survival. As *EGFR* mutations seem to be most common in the classic subtype, this would imply that they might be associated with more favorable outcomes. Case 7 from this study, however, would seem to argue against a simple relationship between the presence of *EGFR* mutations in these tumors and outcomes.

In summary, although the correlation of histology and molecular changes is not absolute, it appears that most if not all classic-type CMNs are driven by *EGFR* mutations, and as we have already known, most cellular-type CMNs are driven by *NTRK3* fusions. The mixed-type CMN represents a more heterogeneous group which may be better defined by molecular changes in the future. There are additional questions that need to be answered. First of all, the biology of *EGFR* KDDs is little studied, although a few studies have shown that they lead to constitutive activation and appear to respond to *EGFR* tyrosine kinase inhibitors in a manner similar to canonical *EGFR* mutations [9,27]. Amplification of the mutated allele has been suggested as a resistance mechanism in the clinical setting [9]. Additional work will be required to determine how much these mutations differ in function from more common activating *EGFR* mutations and whether tyrosine kinase inhibitors will be clinically useful. Another interesting question is whether *EGFR* KDD may be present in other entities showing recurrent *ETV6-NTRK3* fusions such as secretory carcinoma of breast and salivary glands [36,37]. Additional studies should investigate this possibility as these variants might have been missed by NGS variant callers.

## CRediT authorship contribution statement

**Li Lei:** Conceptualization, Methodology, Investigation, Writing - original draft, Writing - review & editing. **Bradley A. Stohr:** Investigation, Resources, Writing - review & editing. **Stacey Berry:** Investigation, Resources, Writing - review & editing. **Christina M. Lockwood:** Investigation, Resources, Writing - review & editing. **Jessica L. Davis:** Investigation, Resources, Writing - review & editing. **Erin R. Rudzinski:** Investigation, Resources, Writing - review & editing. **Christian A. Kunder:** Conceptualization, Writing - review & editing, Methodology, Investigation, Writing - review & editing, Supervision.

## Declaration of competing interest

C. Kunder’s spouse is an employee and shareholder of Genentech. The other authors have no significant relationships with, or financial interest in, any commercial companies pertaining to this article.
